# Palliative care in South Asia: a systematic review of the evidence for care models, interventions, and outcomes

**DOI:** 10.1186/s13104-015-1102-3

**Published:** 2015-04-30

**Authors:** Taranjit Singh, Richard Harding

**Affiliations:** Department of Medical Oncology & Haematology, Artemis Health Sciences Institute, Sector 51, Gurgaon, Haryana 122001 India; Department of Palliative Care, Policy & Rehabilitation, King’s College London, Cicely Saunders Institute, London, UK

**Keywords:** Palliative care, Systematic review, End-of-life, Terminal, Asia, South Asia, Palliative care models

## Abstract

**Background:**

The increasing incidence of cancer and chronic diseases in South Asia has created a growing public health and clinical need for palliative care in the region. As an emerging discipline with increasing coverage, palliative care must be guided by evidence.

In order to appraise the state of the science and inform policy and best practice in South Asia this study aimed to systematically review the evidence for palliative care models, interventions, and outcomes.

**Results:**

The search identified only 16 articles, reporting a small range of services. The 16 articles identified India as having greatest number of papers (n = 14) within South Asia, largely focused in the state of Kerala. Nepal and Pakistan reported a single study each, with nothing from Bhutan, Afghanistan, Maldives or Bangladesh. Despite the large population of South Asia, we found only 4 studies reporting intervention outcomes, with the remaining reporting service descriptions (n = 12).

**Conclusions:**

The dearth of evidence in terms of palliative care outcomes, and the lack of data from beyond India, highlight the urgent need for greater research investment and activity to guide the development of feasible, acceptable, appropriate and effective palliative care services. There is some evidence that suggests implementation of successful and well-developed community based models of palliative care may be replicated in other resource limited settings. Greater investigation to determine outcomes and costs are urgently needed, and require well-designed and validated tools to measure outcomes. Studies are also needed to better understand the cultural context of death and dying for patients and their families in South Asia, and to respond to the growing need for palliative and end-of-life care in the region.

## Background

The World Bank defines the subcontinent of South Asia as Afghanistan, Bangladesh, Bhutan, India, Maldives, Nepal, Pakistan and Sri Lanka (also known as the South Asian Association of Regional Cooperation Countries, or SAARC). It has a combined population of about 1.65 billion, or almost a quarter of the total of the world‘s population, and all SAARC countries are classified as lower middle or low-income countries [1]. An estimated 571 million people in South Asia survive on less than USD 1.25 a day, and they comprise more than 44% of the developing world’s poor (GNI per capita 1,483 USD, 2013) [1].

The International Agency for Research on Cancer (ICAR) estimated the number of new cancer cases annually in South Asia to be around 1.33 million cases (1 million in India, 148,000 in Pakistan, 122,700 in Bangladesh; Nepal 18,800, Sri Lanka 23,700, Afghanistan 20,000, Bhutan 500 and Maldives 200) [2]. In India, more than 80% of cancer patients present at stages 3 and 4 [3]. In addition to late presentation, other factors that increase the need for palliative care include inadequate diagnostic facilities and assessment skills; poor availability of chemotherapy and radiotherapy; and absence of opioids [4]. GLOBOCAN, estimated that there were 919,400 cancer deaths in the year 2012 in South Asia [2,5]. The most recent UNAIDS country data (2012) estimates between 2 and 2.5 million people living with HIV and AIDS in South Asia, and 150,000 HIV-related deaths [6]. Incidence of other chronic diseases, including end-stage heart failure, renal and respiratory diseases, are projected to rise [7-9]. The field of palliative care global health is gaining greater attention, aiming to establish appropriate, locally relevant, feasible and effective palliative care for all irrespective of diagnosis, place of care or geographical region [10]. The most recent global classification of palliative care provision found no evidence of palliative care provision in 19 Indian states and union territories [11]. India and Nepal are categorized under Group 3b, defined as having ‘generalised palliative care provision’. Pakistan, Bangladesh and Sri Lanka are categorized to Group 3a, with ‘isolated palliative care provision’. Afghanistan, Bhutan and Maldives are categorized as Group 1, as there is no known hospice or palliative care activity [12].

The implementation of the WHO’s public health approach to palliative care focuses on education, drug availability, policy and implementation [13], but in addition requires local evidence to underpin strategic development of this strategy [14]. Other low income regions (principally sub-Saharan Africa) have used systematic reviewing of the state of palliative care evidence in order to identify and appraise the existing evidence base [15,16], to highlight the required direction in order to achieve quality coverage for all [17], and subsequently to rapidly grow the evidence base [15,18-22]. In order to catalyse evidence-based policy, funding and practice, and to identify evidence gaps, the aim of this systematic review was to identify and appraise the existing evidence for palliative care models, interventions and outcomes in South Asia.

## Methods

The study implemented a systematic literature review in line with PRISMA guidance [23].

### Search strategy

The following databases were searched in July 2013: Ovid MEDLINE® (1980–2013), PsycINFO (1980–2013), EMBASE (1980–2013) are presented in Table 1 search strategy. The search was updated in Feb 2014, and hand searches were conducted of the *Journal of South Asian Development* and *Indian Journal of Palliative Care.* Reference lists from retrieved articles were subsequently hand searched.

**Table 1:** **Search strategy**

**#**	**Searches**	**Results**
1	hospice.mp. [mp = ti, ab, sh, hw, tn, ot, dm, mf, dv, kw, nm, kf, ps, rs, an, ui, tc, id, tm]	27026
2	terminal.mp. [mp = ti, ab, sh, hw, tn, ot, dm, mf, dv, kw, nm, kf, ps, rs, an, ui, tc, id, tm]	791480
3	terminal care.mp. [mp = ti, ab, sh, hw, tn, ot, dm, mf, dv, kw, nm, kf, ps, rs, an, ui, tc, id, tm]	44526
4	terminally ill.mp. [mp = ti, ab, sh, hw, tn, ot, dm, mf, dv, kw, nm, kf, ps, rs, an, ui, tc, id, tm]	22478
5	palliat*.mp. [mp = ti, ab, sh, hw, tn, ot, dm, mf, dv, kw, nm, kf, ps, rs, an, ui, tc, id, tm]	163014
6	hospice*.mp. [mp = ti, ab, sh, hw, tn, ot, dm, mf, dv, kw, nm, kf, ps, rs, an, ui, tc, id, tm]	29311
7	dying.mp. [mp = ti, ab, sh, hw, tn, ot, dm, mf, dv, kw, nm, kf, ps, rs, an, ui, tc, id, tm]	82104
8	end of life.mp. [mp = ti, ab, sh, hw, tn, ot, dm, mf, dv, kw, nm, kf, ps, rs, an, ui, tc, id, tm]	31866
9	advanced disease.mp. [mp = ti, ab, sh, hw, tn, ot, dm, mf, dv, kw, nm, kf, ps, rs, an, ui, tc, id, tm]	30523
10	life-limiting.mp. [mp = ti, ab, sh, hw, tn, ot, dm, mf, dv, kw, nm, kf, ps, rs, an, ui, tc, id, tm]	1977
11	life-threatening.mp. [mp = ti, ab, sh, hw, tn, ot, dm, mf, dv, kw, nm, kf, ps, rs, an, ui, tc, id, tm]	122999
12	death.mp. [mp = ti, ab, sh, hw, tn, ot, dm, mf, dv, kw, nm, kf, ps, rs, an, ui, tc, id, tm]	1327164
13	bereavement.mp. [mp = ti, ab, sh, hw, tn, ot, dm, mf, dv, kw, nm, kf, ps, rs, an, ui, tc, id, tm]	19321
14	1 or 2 or 3 or 4 or 5 or 6 or 7 or 8 or 9 or 10 or 11 or 12 or 13	2372812
15	Asia.mp. [mp = ti, ab, sh, hw, tn, ot, dm, mf, dv, kw, nm, kf, ps, rs, an, ui, tc, id, tm]	148258
16	South-Asia.mp. [mp = ti, ab, sh, hw, tn, ot, dm, mf, dv, kw, nm, kf, ps, rs, an, ui, tc, id, tm]	4821
17	SAARC.mp. [mp = ti, ab, sh, hw, tn, ot, dm, mf, dv, kw, nm, kf, ps, rs, an, ui, tc, id, tm]	39
18	India.mp. [mp = ti, ab, sh, hw, tn, ot, dm, mf, dv, kw, nm, kf, ps, rs, an, ui, tc, id, tm]	228512
19	Pakistan.mp. [mp = ti, ab, sh, hw, tn, ot, dm, mf, dv, kw, nm, kf, ps, rs, an, ui, tc, id, tm]	33005
20	Bangladesh.mp. [mp = ti, ab, sh, hw, tn, ot, dm, mf, dv, kw, nm, kf, ps, rs, an, ui, tc, id, tm]	20922
21	Afghanistan.mp. [mp = ti, ab, sh, hw, tn, ot, dm, mf, dv, kw, nm, kf, ps, rs, an, ui, tc, id, tm]	9927
22	Nepal.mp. [mp = ti, ab, sh, hw, tn, ot, dm, mf, dv, kw, nm, kf, ps, rs, an, ui, tc, id, tm]	13672
23	Sri Lanka.mp. [mp = ti, ab, sh, hw, tn, ot, dm, mf, dv, kw, nm, kf, ps, rs, an, ui, tc, id, tm]	11947
24	Bhutan.mp. [mp = ti, ab, sh, hw, tn, ot, dm, mf, dv, kw, nm, kf, ps, rs, an, ui, tc, id, tm]	733
25	Maldives.mp. [mp = ti, ab, sh, hw, tn, ot, dm, mf, dv, kw, nm, kf, ps, rs, an, ui, tc, id, tm]	376
26	15 or 16 or 17 or 18 or 19 or 20 or 21 or 22 or 23 or 24 or 25	415069
27	14 and 26	20649
28	limit 27 to english language	18910
29	limit 28 to human	17046
30	limit 29 to yr = “1980 -Current”	16470

Database(s): **Embase** 1980 to 2013 Week 33**, Ovid MEDLINE(R)** 1980 to August Week 3 2013**, PsycINFO,** 1806 to September week 1 2013.

See search terms and inclusion exclusion criteria subsection below:

### Search terms:

#### The union of the following keywords:

- (hospice, terminal, terminal care, terminally ill, palliat*, hospice*, dying, end of life, advanced disease, life-limiting, life- threatening, death, bereavement,)

#### intersected with the union of the following keywords

- (Asia, South Asia, SAARC, India, Pakistan, Bangladesh, Afghanistan, Nepal, Sri Lanka, Bhutan, Nepal and Maldives)

### Inclusion/exclusion criteria

#### Inclusion

Data on care for human subjects

Reported in English language

Peer reviewed journal publication

Data from at least one of the SAARC countries

Data reporting palliative care models (delivery, organisation or content), interventions or outcomes using any study design from at least one of the following settings: home or community care; terminal care; inpatient care; daycare; hospital/acute settings; hospice care; primary care; nursing home; professional or volunteer/lay services; private or government tertiary services; cancer centers; governmental or non-governmental provision; daycare; domiciliary services;

#### Exclusion

Case studies, commentaries, editorials and case reviews

Grey literature

Heterogeneous samples that did not disaggregate patients under palliative care.

### Data extraction and analysis

The search was conducted by TS, and the appraisal of articles against inclusion/exclusion criteria agreed with RH. Data were extracted from the retained papers and entered into common tables. The common data extraction headings were country, aims, methods, sample, service description (with subheadings of structure, provision, activity and funding, each populated according to available information), findings, lessons and comments. This enabled aims, models, study designs and findings to be potentially compared. Once the search was conducted, a post-hoc decision was made not to apply quality criteria or to conduct meta-analysis due to the heterogeneity of aims and designs, and low volume of outcome data.

## Results

**Figure 1:**
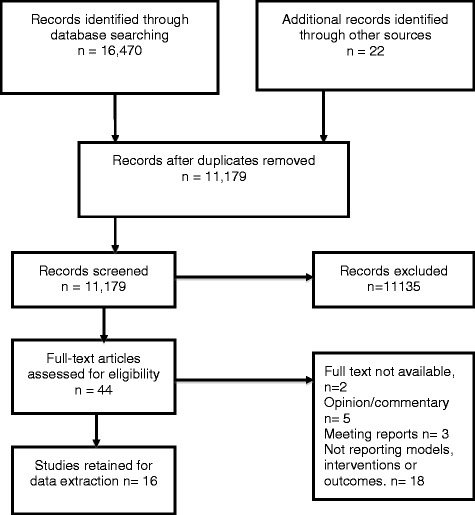
PRISMA flow chart of search strategy.

The papers yielded by the search strategy are reported using the PRISMA flow chart in Figure 1. A total of 16 studies were retrieved and met the inclusion criteria.

The data extraction findings are reported in Table 2.

**Table 2:** **Findings: evidence of palliative care models, interventions or outcomes from south Asia**

**Author& Year, Country, Facility, Reference**	**Service/intervention**	**Aim, methods, sample**	**Findings**	**Conclusions**
**Care models (descriptive)**				
Ajithakumari *et al.,* 1997 *India.* The Pain and Palliative Care Society of Calicut [24]	*Structure*:	Descriptive only (first year of operation).	N/A	N/A
	One doctor with active participation of trained community volunteers.			
	*Provision:*			
	Free community-based outpatient clinics, home care service.			
	*Activity:*			
	3 to 4 visits per day for 2 days a week.			
Seamark *et al.,* 2000, *India.* Models of care across India [25]	*Inpatient care units:*	Descriptive only	N/A	N/A
	a) Hospices			
	Shanti Avedana Ashram, branches Mumbai, Delhi and Goa.			
	b) Government Regional Cancer Centres:			
	11 Government Regional Centres. Few focus on symptom relief:			
	-Regional Cancer Centre, Trivandrum, Kerala.			
	-Palliative Care Centre, Calicut, Kerala.			
	-Pain clinic at Kidwai Memorial Institute of Oncology, Bangalore.			
	*Domiciliary Services:*			
	-Found in Bangalore, Calicut and Delhi cities.			
	*Combined Inpatient and training centre;*			
	-Cipla Cancer and Palliative Care Training Centre, Pune, funded by pharmaceutical company.			
	*Palliative Care education centre;*			
	-Calicut Center			
	-Shanti Avedna Ashram, Mumbai.			
Rajagopal and Palat, 2002, *India.* Models of Palliative Care in Kerala A) Pain and Palliative Care Society (PPCS)- B) Palliative Care Patient’s Benefit Trust (PCPBT)C) Wayanad Palliative Care Consortium (WPCC) [26]	A) PPCS	Descriptive:	N/A	N/A
	*Provision*			
	Outpatient clinic, home visits and inpatient care, educational programs (certificate and diploma programs).			
	*Activity*:			
	27 districts of Kerala via out reach link clinics.			
	B) PCPBT			
	*Provision*			
	Rehabilitation of patients and families, their children education support. Also provide financial support for those who lost livelihood due to disease.			
	C) WPCC			
	*Structure*			
	Regional cooperation model between Govt. hospital, Church and Hindu religious organization.			
Bollini *et al.,* 2004 *India.* Pain and Palliative Care Society (PCCS) [27]	*Structure*	Descriptive only	N/A	N/A
	Free of charge community-based services			
	*Provision*			
	Outpatient clinics, supportive home care services, rehabilitation, health professionals’ training, active participation of trained community volunteers.			
	Most centres licensed to keep oral morphine.			
	*Activity*			
	In 2002, 33 clinics seeing 2000 new patients			
	*Funding*:			
	Private donations and international donors.			
Paleri & Numpeli, 2005, *India.* Models of palliative care in North Kerala [28]	*Structure:*	Descriptive only	N/A	N/A
	Volunteers raise funds; provide social, spiritual and financial support to patients; organise rehabilitation programme.			
	*Provision:*			
	100 palliative care services in the India with 65 centres in Kerala. 57 belong to Neighbourhood Network in Palliative			
	Care (NNPC).			
	20 palliative care units			
	40 home care programme			
	*Activity:*			
	350 home visits /week			
	Trained 3000 volunteers.			
	*Finance:*			
	90% funds raised by local community through donations.			
	*Referral criteria:*			
	Cancer, HIV/AIDS, paraplegia, stroke, old age and debility, psychiatric illness and chronic airway disease.			
Kumar, 2007, *India*. Neighborhood Network in Palliative Care” (NNPC) [29]	*Structure*:	Descriptive only: services/ component offered.	N/A	N/A
	Network to empower local community volunteers to identify and provide long term care and palliative care. More than 60 units covering population around 12 million			
	*Provision:*			
	Regular psychosocial and spiritual support. Home care with outpatient clinical and inpatient units in support. Identifying financial problems, patients in need of care. Create awareness in the community.			
	*Activity:*			
	4000 volunteers, 36 doctors and 60 nurses taking care of approx. caseload of 5000 patients.			
	Volunteer training −16 hours theory session + 4 days clinical training under supervision.			
	*Funding*			
	90% funds raised locally.			
Brown *et al*., 2007, *Nepal* Collaboration of Nepalese International Network for Cancer Treatment and Research (INCTR) and Nepal Palliative Care Group. Collaboration [30]	*Structure:*	Descriptive	N/A	N/A
	Hospice Nepal: 10 bedded, provides home care services, professional education			
	Kanti Children’s Hospital: sole paediatric palliative care service in Nepal. 2 beds for terminally ill.			
	Scheer Memorial Hospital: outreach programme to care patients in rural regions, conduct education programme.			
	Bhaktpur Cancer Hospital: 5 inpatient palliative care beds for, outpatient clinics 2 days/week. 24-hour phone helpline, counselling service.			
	B.P Koirala Memorial Cancer Hospital: Hospice service, home-based care to terminally ill patients including HIV.			
	*Joint activity*:			
	Education and training for professionals, development of clinical guidelines.			
McDermott *et al.*, 2008, *India.* Palliative home care services in India [31]	*Kerala*: PPCS, NNPC	Aims:	138 organizations providing hospice and palliative care services in 16 states and union territories. Concentrated in large cities with the exception of Kerala (n = 63).	Barriers to development include: poverty, population density, geography, opioid availability, workforce development, and limited national policy.
	*New Delhi* **:** CANSupport	1-Systematic overview of current palliative care services across the India	No provision in 19 states/union territories.	Western concept of hospice and palliative care is reshaped to suit the diverse local economic, social and cultural needs.
	*Assam* **:** Guwahati Pain and Palliative Care Society (GPPCS):	2-Identify strengths and weaknesses in palliative care development	Nongovernmental organizations, public and private hospitals, hospices are main providers.	
	*Structure:*	Methods:		
	Volunteer-based	-synthesis of peer review and grey literature		
	*Provision*:	-ethnographic field visits		
	Outpatient clinic, home-care service	-qualitative interviews n = 87 palliative care experts from 12 states		
	*Coverage*:	-collation of existing public health data		
	3 towns in Assam (Rangia, Digboi, and Hojai)			
Banerjee, 2009, *India*. CANSupport Home based palliative care for terminal cancer patients [32]^.^	*Structure:*	Evaluation of effectiveness of homecare teams visit in terminal cancer patients (palliative care).	N/A	
	10 home care teams, each with doctor, nurse and counsellor.	Only presents service descriptive data. .		
	Community network officials, administrative staff			
	*Provision*			
	Home visiting, psychological support, bereavement visit, medicines aid.			
	Telephone helpline active for 8 hours/day for 5 days a week.			
	*Activity:*			
	Total patients seen by homecare teams in 2008–2009 were 1025. 104 patients were discharged. Each team travels 50–150 km per day. 4**–**7 home visits/day by team. First visit- approx. 90–120 minutes. Subsequent visits 30–45 minutes. Usual 1**–**2 months under care.			
Sallnow *et al.,* 2009, *India.* Neighbourhood Network in Palliative Care (NNPC) [33]	*Structure*:	Descriptive: components of NNPC	N/A	N/A
	Home-based model of palliative care in 14 districts of Kerala, 230 clinics, 60-full time doctors and 150 staff nurses, 200 auxiliary nurses and 10,000 trained volunteers			
	*Provision*			
	Home care, outpatient clinics and in-patient services at Institute of Palliative Medicine (IPM) and private hospital free of charge. Medical and nursing care, spiritual and psychological care, medications, training of family members.			
	*Activity*			
	2500 patients/week			
	*Referral* **:**			
	End stage, non-malignant conditions (50%)Cancer patients (30%), HIV/AIDS, chronic psychiatric and problems related to old age			
	*Funding*			
	Raised by local community, small donations from community, government of Kerala and some international agencies.			
Shad *et al.,* 2011, *Pakistan.* A) Shaukat Khanum Memorial Cancer Hospital and Research Center B) Aga Khan University Hospital in Karachi C) Paediatric palliative care [34]^.^	A) *Structure:*	Descriptive only	N/A	N/A
	Palliative-care physician and nurses.			
	*Provision:*			
	Inpatient care, outpatient clinics, 24-hour telephone helpline, pain management, training for physicians*.*			
	B) *Structure:*			
	Palliative care physician, nurse and social worker			
	*Provision*:			
	Inpatient, outpatient service and home care as well, training seminars			
	C) Children Cancer Hospital, Karachi and Children Hospital, Lahore,			
	*Structure:* small inpatient units.			
Kumar, 2013, *India* Kerala State model of palliative care [35]	*Structure:* 90% of all palliative care programs are in state of Kerala, which constitutes 3% of the total population of India. Incorporation of palliative care in the primary healthcare system and public health model initiated by National Rural Health Mission (NRHM) with the palliative care policy of government of Kerala	Descriptive	N/A	Awareness achieved through civil society organizations, media and by NRHM. Decentralized system of governance in Kerala enabled palliative care provision.
	*Provision:*			
	Medical and nursing services like outpatient clinics home care service by volunteers, nurses and doctors Regular supply of food for needy families.			
	Support for children from families of poor patients to continue their education.			
	Transport facilities to referral hospitals.			
	Rehabilitation. Psychological support by trained volunteers. Awareness campaign through local media.			
	*Funding:*			
	State funding by ministry of health, NRHM, and local self-government**.**			
**Outcomes (evaluation data)**				
Bisht *et al.,* 2010, *India.* Evaluation of QOL and pain as an outcome variable of palliative care in advanced cancer patients [38]^.^	*Structure:*	*Aim:*	T0 N = 100	Within palliative care, pain management is key in improving quality of life of advanced cancer patients.
	Oncology clinics of a tertiary teaching hospital.	To evaluate the outcome of palliative care in terms of quality of life and pain control.	T1 N = 93	
	*Provision:*	*Study design:* Observational prospective Study with 2 month follow-up.	T2 N = 51	
	Pain management, palliative chemotherapy, surgery and radiotherapy.	N = 100, mean age 52.57 years.	T0 62% reported pain	
	Home care.	*Measures:*	T1 3%	
		Visual 10 point analogue scale (unspecified).	T2 1%	
		The City of Hope Medical	Reduction in pain	
		Centre Quality of Life survey.	VAS scores (mean ± SD) in from T0 to T1 [7.13 ± 2.2 vs.2.62 ± 2.1 (p < 0.001)].	
			Improvement in	
			the QOL scores [919.78 ± 271.3 vs. 1280.65 ± 306.8(p < 0.01)]. At T2 1405.49 ± 368.3(p < 0.01)	
			Moderate correlation between pain intensity and quality of life scores(r = 0.53, p < 0.001).	
Santha, 2011 *India*. Pain and Palliative care units (PPC), Ernakulum district, Kerala, home care services [36].	*Structure:*	*Aim:*	52% were men (age > 60 yrs)	
	22 units, of which 15 offer home care service.	“Impact” study	50% beneficiaries are cancer patients	
		*Design:*	Major findings:	
		50 patients randomly selected from 15 palliative care units.	Significant difference in types of physical problems faced by the patients(Chi-square = 345.495 p = 0.01).	
		*Study design:* Retrospective descriptive survey	Pain most common	
		*Measures:*	Also ranked highly: social problems;	
		Primary data for descriptive survey with structured questionnaires from the respondents.	not able to stay in job; financial problems/medical bills	
		The study period: 6 months, from July 2009 to January 2010	Major benefit of palliative care sig reduction of pain scores.	
Dongre *et al.,* 2012, *India.* Help Age India, rural Tamil Nadu [39].	*Structure:*	*Aim:*	At palliative care programme entry physical quality of life in intervention area =10.47 ± 1.80 SD compared to control 10.17 ± 1.82 SD (p = 0.013); for psychological support 10.13 ± 2.25 SD vs 9.8 ± 2.29 SD (p = 0.043). Programme shows no effect on domain of social relationship and environment.	Affordable and effective rural palliative care for elderly population at the village level can be can be set up effectively through and community participation.
	Community managed palliative care programme in villages of rural Tamil Naidu state-	To evaluate rural palliative care for older people in terms of quality of life		
	*Provision:*	*Study Design:* Prospective cohort with control comparison group.		
	Home visits by doctor, volunteer, nurse and physiotherapist. Support from Palliative care programme: Home care, Support to buy drugs, rehabilitation support, food, health education, and referral services.	*Sampling:*		
		Project area (n = 450)		
		Control area (n = 450)		
		N = 50 elderly persons, age >60 years in 46 villages		
		Control = 47 neighbouring villages.		
		*Measure:*		
		WHO-Quality of Life-brief questionnaire.		
		*Follow up period of*		
		*study:* From year 2007–2008.		
Thayyil & Cherumanalil, 2012, *India*. Local self-government (Panchayats) led community-based home palliative care [37]^.^	*Structure:*	*Aim:*	*Diagnoses/needs:* 41% degenerative disease, 15.3% malignancies, 13.5% geriatric without any specific diagnosis.	The evaluation concludes that the service could address most of the medical, psychosocial, and supportive needs of the patients and reduce their
	Nurse, health volunteer, social health activist, community member, health department field worker conduct home visits.	To assess patients’ status and services provided	Motor dysfunction (41.3%) tiredness (31.7%) and pain (27%), urinary symptoms (25%), bedridden (25%), ulcer (12.5%), oedema (10.6%), tube feeding (5.8%), urinary incontinence (16.3%), bowel control (9.6%)	pain and symptoms. No change data reported.
	*Provision:*	*Study design:*	Social needs were high with 66.3% receiving cash or material support	
	Medical supportive care, ulcer care, catheter services and supply of accessories	Retrospective record review 2010-2011	Mean duration of care 7.8 ± 5.7 months.	
		n = 104.	36.5% died during period of study.	
		*Measures:* Data on patient problems and time under care extracted.		

### Summary of aims and countries of origin

Of the 16 articles retained, 12 reported service description [24-35], 2 reported service with evaluation data [36,37] and 2 reported outcomes [38,39]. 1 article aimed to collect evaluation data, but actually presented only descriptive data [32]. 14 articles reported data from India, 1 from Nepal [34] and 1 Pakistan [30]. No articles were found originating from Afghanistan, Sri Lanka, Bhutan, Maldives, or Bangladesh. Of these 16 papers, the first was published in 1997, and 6 were published during 5 years prior to the search.

#### Service descriptions

The 11 service descriptions addressed a diversity of care models including home care, cancer centres, hospital consult teams and outpatient clinics. The teams were largely multi-professional, and addressed holistic care needs with an emphasis on rehabilitation and socioeconomic support. The Kerala model is well described in the literature, with a strong community participation approach. Importantly, two pediatric palliative care services were described [30,34].

### Methodological designs & findings

The four papers reporting service evaluations or outcomes used the following designs: a retrospective survey [36]a retrospective file review [37] a prospective longitudinal cohort [38], and a prospective cohort with control comparison group [39] and (as stated above, one paper described as an evaluation only provided service description data). No (quasi) experimental designs were identified.

In terms of the findings, significant improvements in self-report pain among cancer patients were reported (although this prospective study lacked a comparison group [38] and improvements in satisfaction and pain relief reported (although this was retrospective, and again had no comparison group [36]. The prospective comparative cohort study of palliative care for older people found perceived physical quality of life and psychological support among elderly persons was significantly better than the control villages [39], and lastly the retrospective file review of patient problems found a high prevalence of multidimensional needs but did not offer change data to support the conclusion that the service controlled these problems [37].

## Discussion

It is notable that despite the large population and epidemiology of cancer and HIV, there is very small evidence base from which to determine optimal models and interventions of care. It is a strength that there is such innovation and diversity of models, and the Kerala model has been well described and lauded as an appropriate model which other regions may usefully replicate. In terms of the evidence of feasibility and acceptability of palliative care, India has the strongest available literature from the South Asia region. Indian services should be recognised for their commitment to development and implementation of care services, and neighbouring SAARC countries should be encouraged and enabled to improve their coverage similarly. The work to date on model development and initial evaluation places India in particular in a position of readiness to move to more robust outcomes-focused research. The data provides some data on identification of needs, change over time within cohorts under palliative care, and importantly has been generated across diverse settings. In order to move to robust (quasi) experimental evaluative study designs appropriate for palliative care populations, [40] an important next step is the provision of appropriate, valid and reliable outcome measures that reflect the needs of patients and families in India facing life-limiting progressive illness. The development of validation of outcome measurement for African palliative has catalysed research activity in palliative care [41,42]. Palliative care research in India is timely, as palliative care services have been shown to be plausible and sustained. The apparently successful Indian public health approach to palliative care in Kerala should be evaluated to identify and share successful strategies and lessons learned. Additional lessons that are worthy of investigation are the strengths, challenges and mechanisms of volunteerism in India, and the financing models of services that are community-funded and supported. In light of the service descriptions of paediatric palliative care, it has previously been identified that little evidence exists on outcomes of such models in low and middle income countries [16], and we would urge a research focus on this specific population.

We have noted that the majority of data have been published from India, and this suggests that they are in the relatively better position to drive forward the research activities in the region. Within India we also recognize disparity, as the papers suggest that most published work has originated in the south, mostly in or around the state of Kerala, where the literacy rate ranks among the highest in the country, and the population growth rate the lowest [43]. The fact that the search found no articles originating from Afghanistan, Sri Lanka, Bhutan, Maldives, or Bangladesh underlines the importance of undertaking work in these countries, where evidence suggests particularly poor access to palliative care and to opioid pain relief [44,45]. Qualitative studies are also needed to better understand the cultural context of death and dying for patients and their families in South Asia.

## Conclusions

In conclusion, the body of evidence for palliative care in South Asian Association of Regional Cooperation countries is not reflective of the size of population in need. In light of the limited resources available for health systems, evidence is even more important to guide appropriate and effective services. Without adequate assessment, the provision of appropriate, evidence-based palliative care is unlikely to occur; the need for well-designed and validated tools to measure outcomes is paramount to advancing palliative care in a region marked by dire need for it.

## Abbreviations

PRISMA: Preferred Reporting Items for Systematic Reviews and Meta-Analyses
SAARC: South Asian Association of Regional Cooperation
GNI: Gross National Income
USD: United States Dollar
ICAR: International Agency for Research on Cancer
UNAIDS: Joint United Nations Programme on HIV/AIDS
HIV: Human Immunodeficiency Virus
AIDS: Acquired Immunodeficiency Syndrome
WHO: World Health Organization
PPCS: Pain and Palliative Care Society
PCPBT: Palliative Care Patient’s Benefit Trust
WPCC: Wayanad Palliative Care Consortium
NNPC: Neighborhood Network in Palliative Care
INCTR: International Network for Cancer Treatment and Research
GPPCS: Guwahati Pain and Palliative Care Society
IPM: Institute of Palliative Medicine
NRHM: National Rural Health Mission
QOL: Quality of Life
VAS: Visual Analogue Scale
PPC: Pain and Palliative Care units
NGOs: Non-Governmental Organizations

## References

[1] The World Bank (2013). World Development Indicators, GNI per Capita, Atlas method.

[2] International Agency for Research on Cancer(IARC). GLOBOCAN 2008 Project. Lyon, France: International Agency for Research On Cancer (IARC)/WHO; 2008. Available at: http://www.iarc.fr/en/publications/pdfsonline/wcr/2008/index.php.

[3] Takiar R, Nadayil D, Nandakumar A (2010). Projections of number of cancer cases in India (2010–2020) by cancer groups. Asian Pac J Cancer Prev.

[4] Payne S, Chan N, Davies A, Poon E, Connor S, Goh C (2012). Supportive, palliative, and end-of-life care for patients with cancer in Asia: resource-stratified guidelines from the Asian Oncology Summit 2012. Lancet Oncol.

[5] Noronha V, Tsomo U, Jamshed A, Hai M, Wattegama S, Baral R (2012). A fresh look at oncology facts on south central Asia and SAARC countries. South Asian J Cancer.

[6] UNAIDS (2012). Regional Fact Sheet 2012.

[7] Bleumink GS, Knetsch AM, Sturkenboom MC, Straus SM, Hofman A, Deckers JW (2004). Quantifying the heart failure epidemic: prevalence, incidence rate, lifetime risk and prognosis of heart failure the Rotterdam study. Eur Heart J.

[8] Halbert RJ, Natoli JL, Gano A, Badamgarav E, Buist AS, Mannino DM (2006). Global burden of COPD: systematic review and meta-analysis. Eur Respir J.

[9] Murray CJ, Lopez AD (1997). Mortality by cause for eight regions of the world: global burden of disease study. Lancet.

[10] Harding R, Higginson IJ (2014). Inclusion of end-of-life care in the global health agenda. Lancet Global health.

[11] IOELC (2006). Internation observatory on end-of-life care country reports.

[12] Lynch T, Connor S, Clark D (2013). Mapping levels of palliative care development: a global update. J Pain Symptom Manage.

[13] Stjernsward J, Foley KM, Ferris FD (2007). The public health strategy for palliative care. J Pain Symptom Manage.

[14] Harding R, Selman L, Powell RA, Namisango E, Downing J, Merriman A (2013). Research into palliative care in sub-Saharan Africa. Lancet Oncol.

[15] Harding R, Karus D, Easterbrook P, Raveis VH, Higginson IJ, Marconi K (2005). Does palliative care improve outcomes for patients with HIV/AIDS? A systematic review of the evidence. Sex Transm Infect.

[16] Harding R, Albertyn R, Sherr L, Gwyther L (2014). Pediatric palliative care in sub-saharan Africa: a systematic review of the evidence for care models, interventions, and outcomes. J Pain Symptom Manage.

[17] International Association for Hospice and Palliative Care (2007). The declaration of Venice: palliative care research in developing countries. J Pain Palliat Care Pharmacother.

[18] Higginson IJ, Finlay I, Goodwin DM, Cook AM, Hood K, Edwards AG (2002). Do hospital-based palliative teams improve care for patients or families at the end of life?. J Pain Symptom Manage.

[19] Higginson IJ, Finlay IG, Goodwin DM, Hood K, Edwards AG, Cook A (2003). Is there evidence that palliative care teams alter end-of-life experiences of patients and their caregivers?. J Pain Symptom Manage.

[20] Qaseem A, Snow V, Shekelle P, Casey DE, Cross JT, Owens DK (2008). Evidence-based interventions to improve the palliative care of pain, dyspnea, and depression at the end of life: a clinical practice guideline from the American College of Physicians. Ann Intern Med.

[21] Temel JS, Greer JA, Muzikansky A, Gallagher ER, Admane S, Jackson VA (2010). Early palliative care for patients with metastatic non-small-cell lung cancer. N Engl J Med.

[22] Smith TJ, Temin S, Alesi ER, Abernethy AP, Balboni TA, Basch EM (2012). American Society of Clinical Oncology provisional clinical opinion: the integration of palliative care into standard oncology care. J Clin Oncol.

[23] Liberati A, Altman DG, Tetzlaff J, Mulrow C, Gotzsche PC, Ioannidis JP (2009). The PRISMA statement for reporting systematic reviews and meta-analyses of studies that evaluate health care interventions: explanation and elaboration. Ann Intern Med.

[24] Ajithakumari K, Sureshkumar K, Rajagopal MR (1997). Palliative home care–the Calicut experience. Palliat Med.

[25] Seamark D, Ajithakumari K, Burn G, Saraswalthi Devi P, Koshy R, Seamark C (2000). Palliative care in India. J R Soc Med.

[26] Rajagopal MR, Palat G (2002). Kerala, India: status of cancer pain relief and palliative care. J Pain Symptom Manage.

[27] Bollini P, Venkateswaran C, Sureshkumar K (2004). Palliative care in Kerala, India: a model for resource-poor settings. Onkologie.

[28] Paleri A, Numpeli M (2005). The evolution of palliative care programmes in North Kerala. Indian J Palliat Care.

[29] Kumar SK (2007). Kerala, India: a regional community-based palliative care model. J Pain Symptom Manage.

[30] Brown S, Black F, Vaidya P, Shrestha S, Ennals D, LeBaron VT (2007). Palliative care development: the Nepal model. J Pain Symptom Manage.

[31] McDermott E, Selman L, Wright M, Clark D (2008). Hospice and palliative care development in India: a multimethod review of services and experiences. J Pain Symptom Manage.

[32] Banerjee P (2009). The effect of homecare team visits in terminal cancer patients: role of health teams reaching patients homes. Indian J Palliat Care.

[33] Sallnow L, Kumar S, Numpeli M (2010). Home-based palliative care in Kerala, India: the neighbourhood network in palliative care. Prog Palliat Care.

[34] Shad A, Ashraf MS, Hafeez H (2011). Development of palliative-care services in a developing country: Pakistan. J Pediatr Hematol Oncol.

[35] Kumar S (2013). Models of delivering palliative and end-of-life care in India. Curr Opin Support Palliat Care.

[36] Santha S (2011). Impact of pain and palliative care services on patients. Indian J Palliat Care.

[37] Thayyil J, Cherumanalil JM (2012). Assessment of status of patients receiving palliative home care and services provided in a rural area-kerala, India. Indian J Palliat Care.

[38] Bisht M, Bist S, Dhasmana D, Saini S (2010). Quality of life as an outcome variable in the management of advanced cancer. Indian J Med Paediatr Oncol.

[39] Dongre AR, Rajendran KP, Kumar S, Deshmukh PR (2012). The effect of community-managed palliative care program on quality of life in the elderly in rural Tamil Nadu, India. Indian J Palliat Care.

[40] Evans CJ, Harding R, Higginson IJ (2013). ‘Best practice’ in developing and evaluating palliative and end-of-life care services: a meta-synthesis of research methods for the MORECare project. Palliat Med.

[41] Harding R, Selman L, Agupio G, Dinat N, Downing J, Gwyther L (2010). Validation of a core outcome measure for palliative care in Africa: the APCA African Palliative Outcome Scale. Health Qual Life Outcomes.

[42] Selman L, Harding R, Gysels M, Speck P, Higginson IJ (2011). The measurement of spirituality in palliative care and the content of tools validated cross-culturally: a systematic review. J Pain Symptom Manage.

[43] Office of Registrar General and Census Commissioner (2011). Chapter 6: State of Literacy. Census of India. Government of India, Ministry of Home Affairs. Provisional Population totals-India, State of Literacy, Chapter 6.

[44] Dehghan R, Ramakrishnan J, Ahmed N, Harding R (2010). The use of morphine to control pain in advanced cancer: an investigation of clinical usage in Bangladesh. Palliat Med.

[45] Office of Registrar General and Census Commissioner. [http://www.censusindia.gov.in/2011-prov-results/data_files/india/Final_PPT_2011_chapter6.pdf]

